# ^18^F-FDG-PET/CT Imaging in Advanced Glottic Cancer: A Tool for Clinical Decision in Comparison with Conventional Imaging

**DOI:** 10.1155/2019/4051206

**Published:** 2019-09-11

**Authors:** G. Paone, F. Martucci, V. Espeli, L. Ceriani, G. Treglia, T. Ruberto, A. Richetti, R. Piantanida, L. Giovanella

**Affiliations:** ^1^Department of Nuclear Medicine and PET/CT Centre, Imaging Institute of Southern Switzerland, Bellinzona, Switzerland; ^2^Department of Radiotherapy, Oncology Institute of Southern Switzerland, Bellinzona, Switzerland; ^3^Department of Medical Oncology, Oncology Institute of Southern Switzerland, Bellinzona, Switzerland; ^4^Department of Otolaryngology-Head and Neck Surgery, Ospedale Regionale di Lugano, Lugano, Switzerland

## Abstract

This study assessed the role of ^18^F-FDG PET-CT (PET/CT) to detect the cartilage and paraglottic infiltration in advanced glottic cancer comparing the results with those of conventional imaging (CI) (contrast-enhanced computed tomography and/or magnetic resonance). In addition, we assessed the prognostic value of quantitative parameters, measured on baseline PET/CT, in terms of event-free survival (EFS) and overall survival (OS). We retrospectively analyzed 27 patients with glottic squamous cell carcinoma stage III and IVA, treated in our institute between 2010 and 2016, comparing PET/CT, performed for staging and radiotherapy planning, and CI findings. Cohen's *K* was used to compare concordance between PET/CT and CI. Imaging findings were correlated with endoscopic evaluation and histological reports (gold standard (GS)). All lesions shown by CI were also detected by PET/CT imaging, and in 5 cases, a better definition of local infiltration was achieved with PET/CT than CI (5 CT). Sensitivity, specificity, and accuracy of PET/CT and CT were 95%, 86%, and 93% and 70%, 86%, and 74% for, respectively. MRI showed sensitivity and specificity of 100%. One false-negative (FN) cases and 1 false-positive (FP) case were observed with PET/CT with no difference compared to MRI (10 cases). Six FN cases and 1 FP case were observed with CT. Cohen's *K* was 0.60 (PET vs. CI) and 0.80 (PET vs. GS). Patients were followed-up for at least 24 months to calculate EFS and OS. 13 local recurrence and 7 deaths were recorded. Among quantitative PET parameters, baseline MTV was the most powerful predictor of outcome. Our data suggest a reliable sensitivity and accuracy of PET/CT in the evaluation of local extension, proving a useful method for initial local staging in addition to the well-established role in lymph-node and distant sites assessment. Furthermore, pretreatment MTV provides better prognostic information than other PET/CT parameters.

## 1. Introduction

Glottic cancer (GC) is a common malignant tumor in the head and neck region. Histologically, more than 95% of these tumors are represented by squamous cell carcinoma (SCC) [1–3]. Staging of primary glottic SCC (GSCC) is based on clinical examination using indirect and direct endoscopy to evaluate larynx involvement or adjacent regions of the pharynx, as well as vocal cord mobility. Additional imaging procedures are required in advanced cases to accurately evaluate the invasion of submucosal tissue and extension into deep planes [4, 7]. T3 lesions have extension into the paraglottic and/or preepiglottic space, irrespective of vocal cord mobility and tumor invasion limited to the inner cortex of the thyroid cartilage. T4a lesions are characterized instead by extralaryngeal tumor spread and invasion through the thyroid cartilage.

Different therapeutic approaches can be considered, depending on the stage: organ preservation treatments as radiation therapy, chemoradiation protocols, or function-preserving partial surgery, namely, for T3 lesions. More aggressive treatments up to total laryngectomy are indicated in patients with T4a disease, particularly when the tumor extends through the cartilage into the soft tissues [5–9]. Conventional imaging (CI) (contrast-enhanced CT (CECT) and MRI) is mandatory to complete the staging process, assessing tumor extent and to detect and define nodal disease. Larynx cancer is frequently evaluated with CECT which is less affected by breathing and swallowing artifacts, while MRI should be preferred for nasopharyngeal, sinonasal, and parotid tumors for a better contrast resolution and detection of perineural spread [10, 11]. ^18^F-FDG PET/CT (PET/CT) instead shows clear advantage, compared to CI, for assessment of subclinical nodal disease, distant metastases, and evaluation of the treatment results [12, 13].

Cartilage invasion plays a crucial role in local staging of GSCC, an important landmark to qualify a T4a stage. The characteristic pattern on CT image is represented by sclerosis and invasion in thyroid, cricoid, and arytenoid cartilage, in combination with extralaryngeal infiltration. In such cases, the MRI findings (i.e., high signal intensity in T2 sequences and a low/intermediate signal in T1) are indistinguishable from those found in reactive inflammation, edema and fibrosis. In addition, MRI is costly and time consuming compared to CT and, as a consequence, the latter is the preferred technique to evaluate the primary site and the possible nodal involvement [10]. On the other hand, PET/CT proved to be more sensitive and accurate in detecting distant metastases and any second tumor and as a prognostic index, especially by using semiquantitative parameters (i.e., standard uptake value (SUV), metabolic tumor volume (MTV), and total lesion glycolysis (TLG)) [14–20]. In addition, promising preliminary result was also reported in locoregional staging, on management of radiotherapy plan, demonstrating an improvement on dosimetry by lowering dose to certain organs at risk, and in comparison with panendoscopy to detect unknown secondary primary tumors [21–24].

In our retrospective study, we assessed the diagnostic accuracy of PET/CT for advanced glottic cancer, considering laryngeal cartilage infiltration rates (sensitivity, specificity, accuracy, negative predictive value (NPV), and positive predictive value (PPV)) and paraglottic infiltration. We eventually have compared PET/CT with CECT and MRI. Histological specimen analyses were used as standard of reference. Finally, we assessed the prognostic value of quantitative parameters, measured on baseline PET/CT, in terms of event-free survival (EFS) and overall survival (OS).

## 2. Materials and Methods

Records of 31 patients with histologically confirmed advanced GSCC (stage T3–T4a) treated between January 2010 and December 2016 in our institute were retrieved and reviewed. 4 patients were excluded for lack of data on follow-up. 27 patients (mean age 67.1 years; range 55–80, 3 females and 24 males) undergoing complete endoscopic workout and surgical treatment (total laryngectomy with or without thyroidectomy) were eventually included. PET/CT and CECT were performed, as staging procedures, within two weeks after panendoscopy and before any treatment [25]. In addition to PET/CT and CECT, a MRI examination was performed in 10 patients.

A nuclear medicine physician and a radiologist specialized in head and neck imaging reviewed PET/CT and CI, respectively, in a double-blind method. Initially, PET/CT data were visually analyzed to identify focal FDG uptake corresponding to the primary lesion that resulted significantly higher than background activity of surrounding tissues (at least 3 times higher) and to evaluate whether FDG-avid area overlapped the laryngeal cartilage framework, using the coregistered low-dose CT images. Fused images were then elaborated on the three orthogonal planes to visualized disease's extension on the surrounding region (supra- and subglottis space) while axial plane was dedicated to assess possible infiltration of laryngeal cartilage and involvement of extralaryngeal tissue. Semiquantitative analysis was performed using a semiautomated circular 3D VOI that allowed to calculate SUVmax, peak, MTV in cm^3^, and TLG (contouring program on Syngo TrueD workstation, Siemens). CECT images were analyzed to define the cartilage involvement based on a combination of invasion, erosion and transmural extralaryngeal spread. Finally, on MRI, Becker's criteria were used to identify the suspected cartilage invasion comparing T2-weighted and contrast-enhanced T1-weighted signal [26–28].

Patients were followed-up for at least 24 months to calculate EFS and OS. Follow-up consisted in head and neck specific examination and exploration of the inner structures using endoscopic procedure (mirror and a flexible endoscope) every 3 months during the first two years. Imaging procedures (PET/CT and eventually contrast-enhanced CT) were performed every year for the first 2 years; subsequent imaging studies were considered in case of clinical suspicion of disease recurrence. EFS was defined as the time from diagnosis to disease progression (relapse or death) and OS as the time from diagnosis to death.

Statistical analysis was carried out using SPSS for Windows. The agreement test (Cohen's Kappa) was used to compare imaging modality with the histological findings (GS). When appropriate, the Fisher test was used and *p* value < 0.05 was considered statistically significant. PET/CT-derived parameters (SUVmax, MTV, and TLG) were analyzed using receiver-operating characteristic (ROC) analysis to identify the optimal cutoff point. The area under the ROC curve (AUC) and the diagnostic accuracy for recurrence and death were used to select the best method. EFS and OS, chosen as endpoints to evaluate prognosis, were estimated using the Kaplan–Meier method. Logistic regression and Cox proportional-hazards regression were performed to test the significance of SUVmax, MTV, and TLG.

## 3. Results

**Figure 1:**
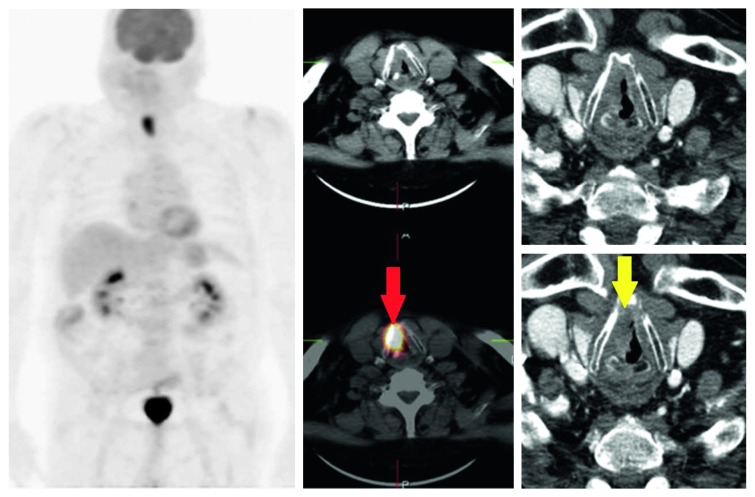
Pt T4a (cartilage infiltration): PET/CT positive (TP, red arrow) vs. CECT negative (FN, yellow arrow). SUVmax, 19.52; MTV, 11.15 cm^3^.

**Figure 2:**
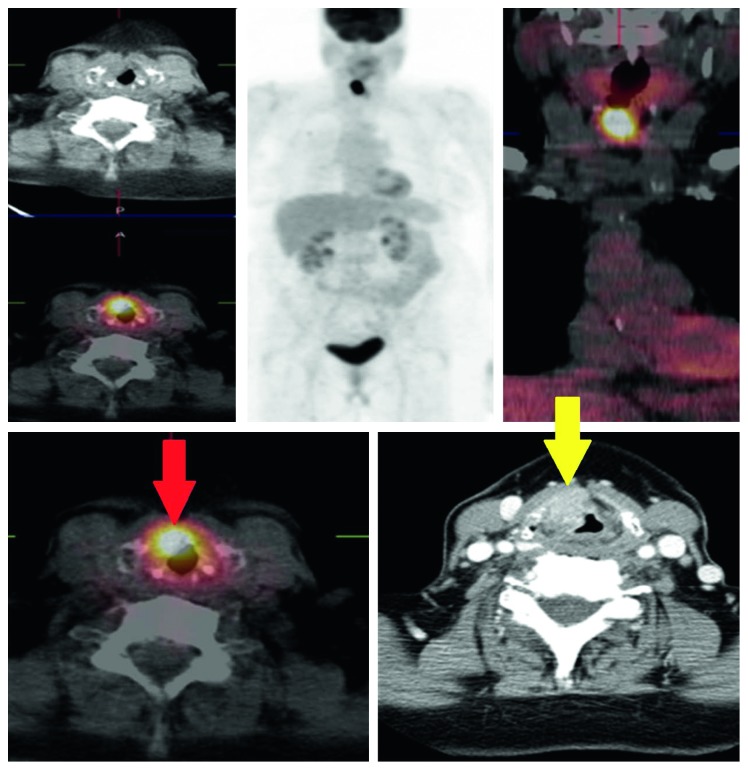
Pt T3 (no cartilage infiltration): PET/CT positive (FP, red arrow) vs. CECT positive (FP, yellow arrow). SUVmax, 19.99; MTV, 8.81 cm^3^.

On the basis of histological findings, our patients were included into two groups: T4a showing infiltration of the cartilaginous structures and extralaryngeal spread (20/27 (74%)) and T3 without cartilage infiltration and tissue invasion (7/27 patients (26%)) (Figures 1 and 2).

As summarized in Table 1, PET/CT and CECT correctly detected cartilage infiltration and extralaryngeal invasion, respectively, in 19 and 14 of 20 patients of the T4a group.

**Figure 3:**
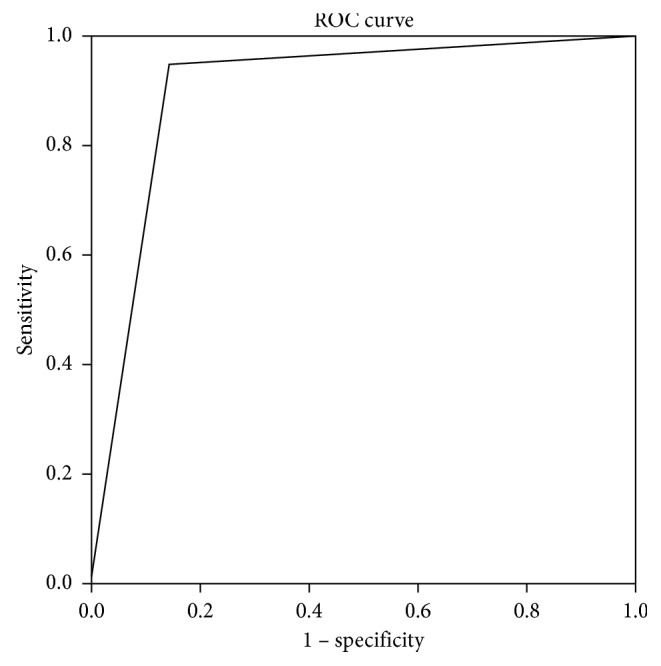
ROC Curve for evaluation of sensitivity and specificity of PET/CT. Infiltration PET: AUC 0.902.

**Figure 4:**
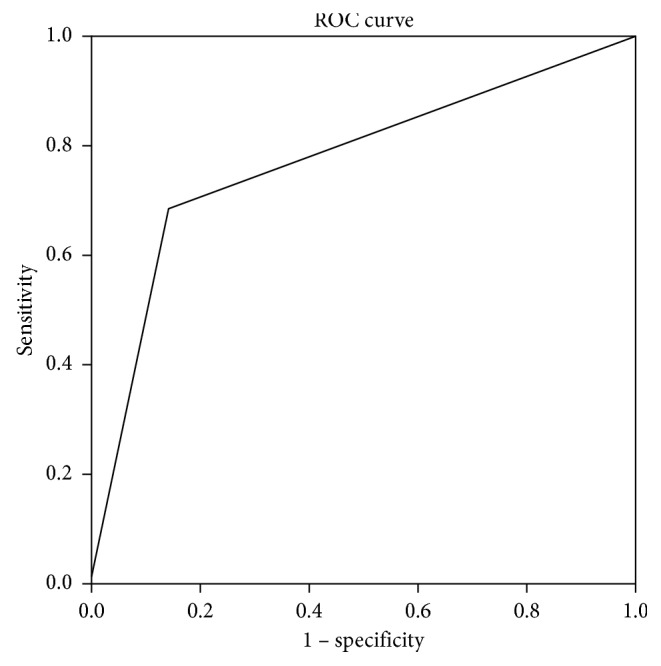
ROC Curve for evaluation of sensitivity and specificity of CECT. Infiltration CECT: AUC 0.771.

Both methods correctly excluded cartilage infiltration and extralaryngeal invasion in 6/7 patients with T3 stage, with one false positive (FP) in the same patient (Figure 2). We found one false negative (FN) in PET/CT and six in CECT. In summary, the sensitivity and specificity for PET/CT were, respectively, 95% and 86% (95% CI: 75% to 99%; 48% to 97%) while for CECT were 70% and 86% (95% CI: 46% to 85%; 49% to 97%). PET/CT had accuracy, PPV, and NPV, respectively, of 93%, 95%, and 86% vs. 74%, 93%, and 50% of CECT. Finally, MRI was also performed in 7 of 20 T4a patients showing sensitivity and specificity of 100% (95% CI 64% to 100%; 44% to 100%). Area under the receiver-operating characteristic curve for cartilage infiltration detected by PET/CT (0.90) was significantly larger than that of CECT (0.77) (*p* = 0.001) (Figures 3 and 4).

Coefficient of agreement between imaging modality and histological findings was excellent for PET/CT (*k* 0.805) and MRI (*k* 1.00) while it resulted moderate for CECT (*k* 0.442). Coefficient of agreement calculated between different imaging methods was excellent for PET/CT vs. MRI (*k* 1.00) and good for PET/CT vs. CECT and MRI vs. CECT (*k* 0.604). No significant differences, in terms of local staging, were found between the two groups using the semiquantitative analysis.

After a median follow-up of 37 ± 10.6 months, 13 local recurrence (within 15 ± 8.5 months) and 7 deaths (within 26 ± 14 months) were recorded.

The optimal SUVmax, MTV, and TLG cutoff values to discriminate subgroups with different event-free (EFS) and overall survival (OS) have been defined by ROC curve analysis (Tables 2 and 3). As summarized in Table 4, we calculated sensitivity (Se.), specificity (Sp.), positive predictive value (PPV), negative predictive value (NPV), and accuracy (Acc.) of these cutoff values to predict recurrence and death.

**Figure 5:**
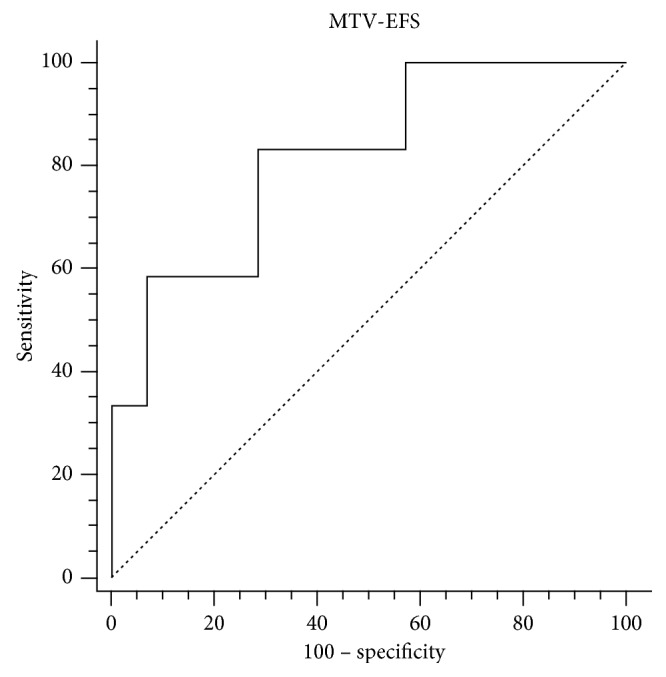
ROC curve MTV-EFS.

**Figure 6:**
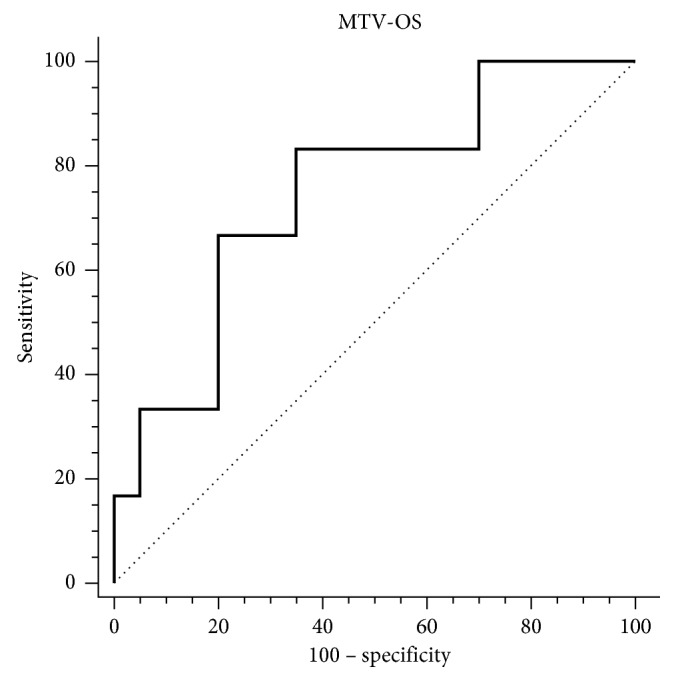
ROC curve MTV-OS.

**Figure 7:**
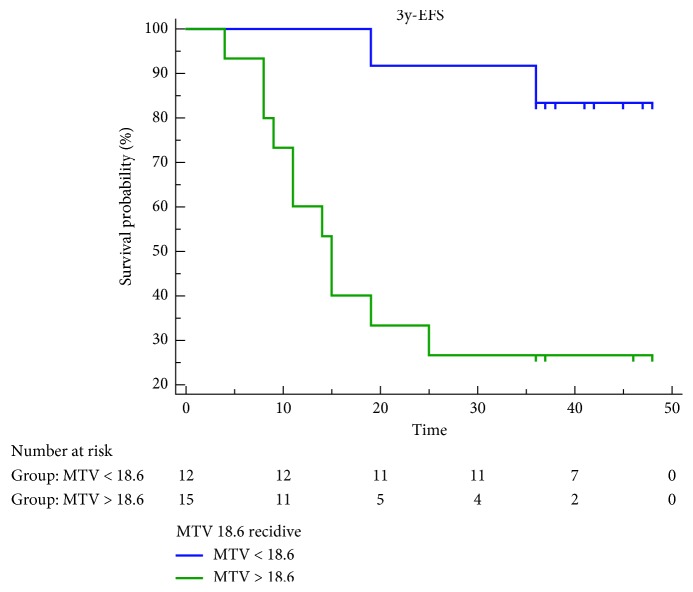
Kaplan–Meier estimates of event free survival (EFS).

**Figure 8:**
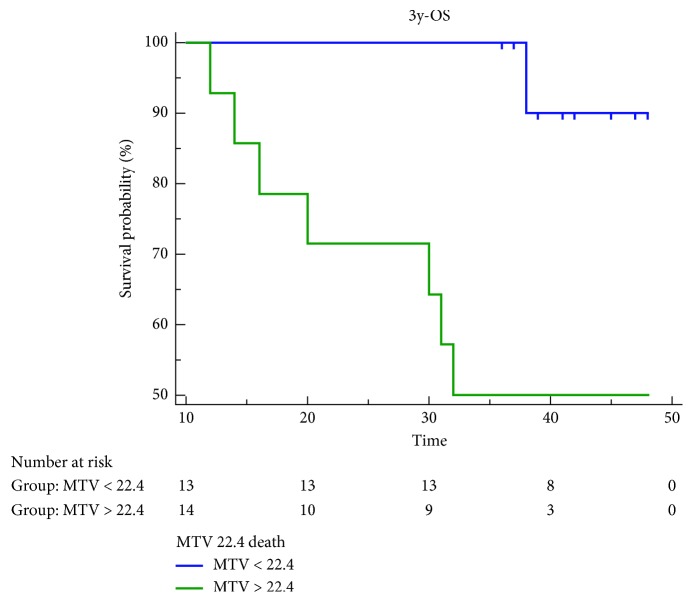
Kaplan–Meier estimates of event free survival (EFS).

High values of MTV, SUVmax, and TLG showed a statistically significant association with worse EFS, but only MTV retained statistical significance for both OS and EFS, yielding the best prediction of recurrence and death (Table 5, Figures 5 and 6). Cox proportional-hazards regression showed that age, sex, staging, and TNM classification were not related to the patients' outcome. There was a significant difference for predicting EFS (*p* < 0.0016; HR 7.3518, 95% CI 2.4505 to 22.0570) and OS (*p* < 0.0092; HR 9.4105, 95% CI 2.3180 to 38.2045) for patients with high MTV (cutoff EFS MTV > 18.6 cm^3^ and OS MTV > 22.4 cm^3^) vs. low MTV. The estimated 3-year EFS (median 44 months 95% CI 39.836 to 49.331) was 83% for patients with low MTV vs. 26% for those with high MTV. 3-year OS (median 47 months 95% CI 45.141 to 48.859) was 92% vs. 50%, respectively (Figures 7 and 8). Global overall survival was 74% (20/27 pts). Long-term outcome was significantly better for patients with lower MTV values compared with those with high MTV values. Among quantitative PET/CT parameters, baseline MTV was the most powerful predictor of outcome.

## 4. Discussion

**Figure 9:**
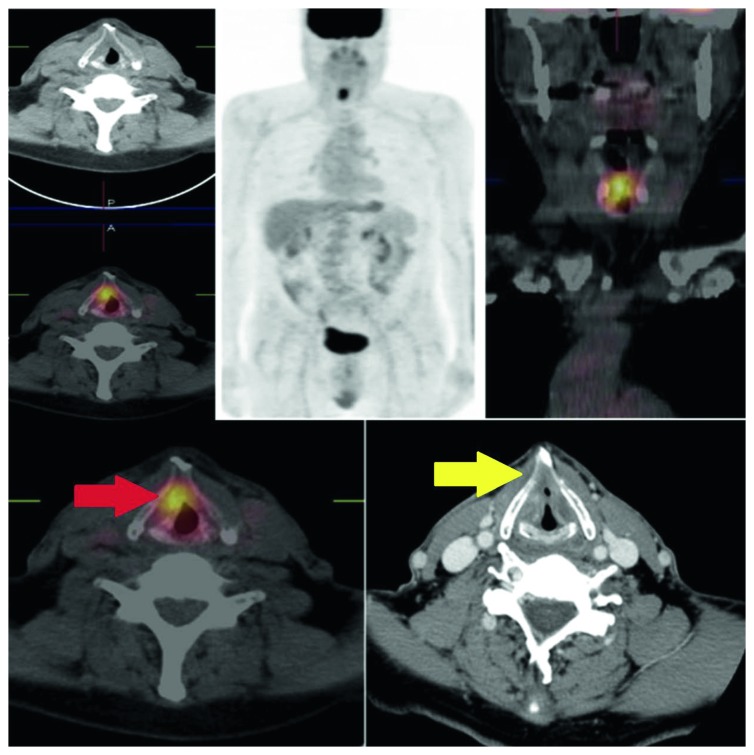
Pt T4 (cartilage infiltration): PET/CT negative (FN, red arrow) vs. CECT negative (FN, yellow arrow). SUVmax, 9.46; MTV, 2.35 cm^3^.

According with the latest treatment guidelines [4], advanced glottic SCC with cartilage invasion and extralaryngeal tissue infiltration favors total laryngectomy followed by radiotherapy and chemotherapy rather than approaches based on organ preservation protocols [29]. Imaging techniques are mandatory in addition to clinical and endoscopic examinations for an appropriate staging of the disease [30]. Several studies highlighted the impact of conventional imaging methods for detection of cartilage invasion in advanced GSCC showing a high sensitivity (94% vs. 67%) and a low specificity (74% vs. 87%) for MRI compared to CECT [27, 31, 32]. Recently, Becker et al. proposed new diagnostic MRI criteria to increase specificity up to 84% without affecting sensitivity [28]. Metabolic imaging by ^18^F-FDG-PET/CT may improve the accuracy of head and neck cancer (HNC) staging, distinguish residual or recurrent disease from posttherapeutic changes, differentiate early responders patients from nonresponders, and provide prognostic data [33–36]. However, so far, there are few published data defining the role of PET/CT on the local staging of advanced glottic cancer and, therefore, selecting the treatment options [16, 37, 38]. Our study provides preliminary data supporting the valuable accuracy of PET/CT compared to CI in detecting cartilage invasion and local infiltration in advanced GSCC. Interestingly, while CECT showed FN results in 6/13 patients, only one FN result occurred with PET/CT series (Figure 9), probably related to a very small metabolic volume of primary tumor.

**Figure 10:**
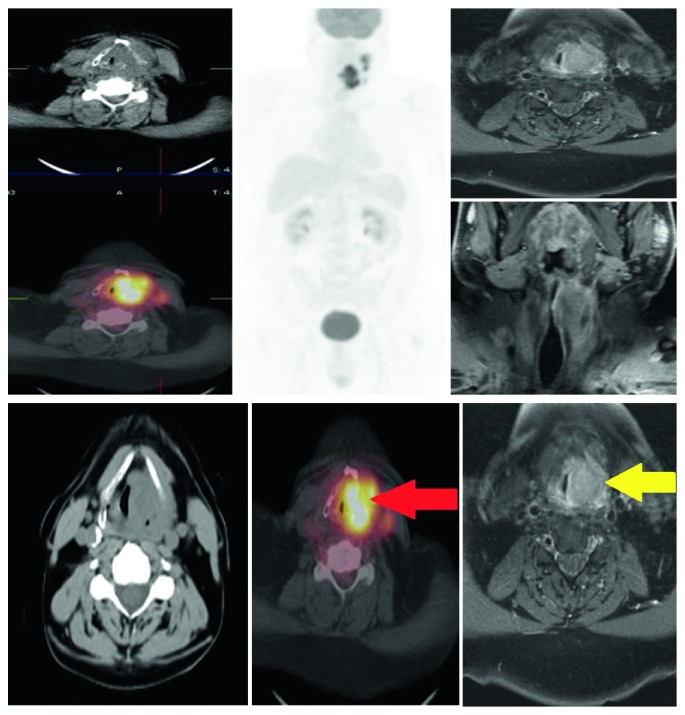
Pt T4 (cartilage infiltration): PET/CT positive (TP, red arrow) vs. MR positive (TP, yellow arrow). SUVmax, 18.56; MTV, 43.03 cm^3^.

No significant differences were found between PET/CT and MRI. Unfortunately, the latter was performed only in 10 patients not including FN and FP cases highlighted in PET/CT (Figure 10). This precludes any conclusion even if current literature data demonstrated similar results of PET/CT and MRI in local staging of head and neck cancers, although different indications are linked to specific anatomical sites [39–44].

Recent studies have also evaluated the role and the diagnostic value of PET/contrast-enhanced CT (PET/CECT) as one step examination with encouraging data, especially during the initial staging, recognizing it as a valid alternative to the usual combination of two separate imaging procedures. The major advantage, over other imaging methods, is the capability to detect small lymph nodes in the neck zone and to provide more accurate anatomical details of primitive lesion. Even in posttreatment phase, especially after surgery, it appears to be the most suitable restaging tool showing a higher accuracy for diagnosing overall recurrence [45–48].

Considering the high-quality anatomic imaging and resolution of neck MRI and the high sensitivity of metabolic imaging with 18FDG, Kuhn et al. indicated that PET/MRI examination might serve as alternative to PET/CT in the clinical workup of patients with head and neck tumors [49]. Platzek et al. demonstrated the feasibility of whole-body PET/MRI, suggesting a potential improvement in cases with soft tissues infiltration [50]. However, a similar diagnostic accuracy of PET/CT and PET/MRI was reported by Kubiessa (i.e., sensitivity, specificity, PPV, and NPV of 82.7, 81.3, 92.4, and 73.2% for PET/CT vs. 80.5, 88.2, 75.6, and 92.5% for PET/MRI) [51]. The potential advantages offered by this new method rely on the better soft tissue contrast on MRI and reduced exposure to radiation compared to PET/CT especially when a CECT is performed.

Anyway, large prospective studies are required to define clinical advantages of PET/MRI, and this new method cannot yet replace PET/CT.

In addition, various quantitative PET/CT-derived parameters have been tested in the pre- and postradiotherapy settings as potential prognostic factors in HNSCC. While SUV was largely studied as PET/CT-derived quantitative parameter, MTV and TLG recently emerged as reliable prognostic marker in solid tumors. In the literature, several studies highlight the possible impact of these prognostic parameter in oropharyngeal and esophageal carcinoma while, so far, there are few published data addressing advanced GSCC [52–56].

MTV and TLG, combining tumor volume and metabolic activity of the entire tumor, are expected to provide a realistic volumetric evaluation of tumor burden with a primary prognostic role in malignant disease [57, 58]. MTV and TLG were introduced in HNC as tools to improve the prognostic stratification and to support other PET/CT-guided radiotherapy plan [18, 19, 23]. These quantitative parameters were analyzed in several series of oropharynx squamous cell carcinoma, and, recently, Wang et al. in a meta-analysis confirmed their superiority compared to SUVmax, highlighting MTV and TLG as prognostic biomarkers to predict outcome in patients with head and neck cancer [59]. Kendi et al. evaluated PET/CT parameters impact to differentiate invasion and penetration of thyroid cartilage highlighting that TLG and MTV had enough power to predict thyroid cartilage invasion and penetration in irradiated patients. In our study, we evaluated, instead, only nonirradiated patients, and above all, in T4a patients, we found extralaryngeal tissues invasion through the thyroid cartilage with infiltration of inner and outer cortices [60].

Considering the data already known in the literature, we analyzed the prognostic value of these parameters in a uniform series of advanced GSCC.

Our results support baseline MTV as the strongest predictor of outcome for patients with advanced GSCC using two different cutoffs to predict EFS (MTV > 18.6 cm^3^) and OS (MTV > 22.4 cm^3^), as described in previous studies focusing on heterogeneous patient series [20, 55].

These data could be extremely useful in patient's stratification, classifying different relapse risks. PET/CT, in addition to the already approved major clinical tool for monitoring treatment in HNSCC, is therefore a diagnostic procedure capable to select patients undergoing staging, directing them to more or less intensive treatments.

## 5. Conclusion

Our data suggest a reliable sensitivity and accuracy of PET/CT in the evaluation of local extension (i.e., cartilage invasion and extralaryngeal tissue infiltration) in advanced GSCC in comparison with CECT. PET/CT is a valuable method for initial local staging in addition to its well-established role for neck nodes and distant metastasis assessment. The present study may provide the basis, in our opinion, for prospective studies on larger series of patients; for these particular tumors, it would also be desirable to compare PET/CT and PET/MRI data.

Furthermore, pretreatment MTV provides better prognostic information than other PET/CT-derived parameters; it can be used as an independent long-term predictive factor in patients with advanced GSCC.

## Figures and Tables

**Table 1:** Primary tumor staging (T) according to the AJCC and summary's results of different imaging modalities (PET/CT vs. CECT vs. MRI).

GSCC (*n* 27): T3 and T4a	PET/CT (*n* 27)	CECT (*n* 27)	MRI (*n* 10)
T4a (*n* 20)	19 TP	14 TP	7 TP
	1 FN	6 FN	
T3 (*n* 7)	6 TN	6 TN	3 TN
	1 FP	1 FP	

TP = true positive; TN = true negative; FP = false positive; FN = false negative.

**Table 2:** ROC curve for EFS.

Parameters	AUC (95% CI)	*p*	Cutoff	Sensitivity (95% CI) (%)	Specificity (95% CI) (%)
MTV	0.815 (0.615–0.939)	0.0002	18.6	83.3 (51.6–97.9)	71.4 (41.9–91.6)
SUVmax	0.764 (0.562–0.905)	0.0057	6.8	100 (75.3–100)	42.8 (17.7–71.1)
TLG	0.799 (0.602–0.928)	0.0005	78.8	84.6 (54.6–98.1)	71.4 (41.9–91.6)

**Table 3:** ROC curve for OS.

Parameters	AUC (95% CI)	*p*	Cutoff	Sensitivity (95% CI) (%)	Specificity (95% CI) (%)
MTV	0.750 (0.543–0.897)	0.0353	22.4	83.3 (35.9–99.6)	65 (40.8–84.6)
SUVmax	0.786 (0.586–0.919)	0.0018	17.3	71.4 (29.0–96.3)	80 (56.3–94.3)
TLG	0.775 (0.574–0.912)	0.0161	124	85.7 (42.1–99.6)	65 (40.8–84.6)

**Table 4:** Diagnostic accuracy of PET/CT-derived parameters' cutoff values.

	MTV 18.6 (EFS) (%)	SUVmax 6.8 (EFS) (%)	TLG 78.8 (EFS) (%)	MTV 22.4 (OS) (%)	SUVmax 17.3 (OS) (%)	TLG 124 (OS) (%)
Se.	85	100	85	86	71	86
Sp.	71	43	71	60	75	65
PPV	73	62	73	43	50	46
NPV	83	100	83	92	88	93
Acc.	78	70	78	67	74	70

**Table 5:** Logistic regression for MTV, SUVmax, and TLG.

Parameters	(95% CI)	OR	*p* (3-year EFS)	(95% CI)	OR	*p* (3-year OS)
MTV	1.0108 to 1.1803	1.0923	0.0022	1.0057 to 1.1271	1.0647	**0.0081**
SUVmax	1.0098 to 1.3185	1.1539	0.0165	0.9619 to 1.2098	1.0788	0.1814
TLG	1.0015 to 1.0205	1.0109	0.0022	0.9903 to 1.0010	1.0588	0.0942

## Data Availability

The dataset generated during the current study and the data used to support the results are available from the corresponding author on reasonable request.
